# Rapid releasing naproxen Liqui-Pellet using effervescent agent and neusilin US2

**DOI:** 10.22038/ijbms.2020.51697.11729

**Published:** 2021-01

**Authors:** Matthew Lam, Kofi Asare-Addo, Ali Nokhodchi

**Affiliations:** 1 Pharmaceutics Research Laboratory, Arundel Building, School of Life Sciences, University of Sussex, Brighton, UK; 2 Department of Pharmacy, University of Huddersfield, Huddersfield, HD1 3DH, UK

**Keywords:** Coating, Excipients, Liqui-Mass system, Rapid release, Solubility, X-ray tomography

## Abstract

**Objective(s):**

Liqui-Mass technology has shown promising advantages in terms of commercial production and formulation manipulation. This study attempts to further explore the potential of enhanced drug release of effervescent Liqui-Pellet by optimizing certain parameters.

**Materials and Methods:**

In the current study, pellets containing co-solvent, naproxen, coating and carrier materials were prepared via extrusion and spheronisation (Liqui-Pellet). Parameters investigated included polysorbate 80 concentration (as a co-solvent), water content and the presence or absence of neusilin US2 as part of the new binary carrier mixture approach.

**Results:**

It was found that the success of the Liqui-Pellet production was determined by the amount of polysorbate 80 and water used, where above a certain limit, agglomeration occurred, and the formulation failed. Liqui-Pellet formulation showed an excellent flow, narrow size distribution and was robust to pass friability testing. The key findings in the investigation were that the Liqui-Pellet was capable of a remarkably fast drug release, and 100% drug release achieved within 20 min at pH 1.2, wherein naproxen has been known to be practically insoluble in such pH.

**Conclusion:**

Liqui-Pellets display the potential to enhance explosive dissolution where a combination of effervescent powders and binary carriers with the high surface area were used. Furthermore, X-ray microtomography revealed that the pellets were very uniform and homogenous.

## Introduction

Liqui-Pellet is a novel oral dosage form, which is believed to have the potential for major contribution to the next generation of oral dosage forms (1–4). Liqui-Pellet emerged from the combination of the concepts from liquisolid with pelletization technologies. It is important to note that Liqui-Pellet differs from the liquisolid pellet (5), which is a liquisolid formulation. Liquisolid pellet uses liquisolid system (6) and is part of the liquisolid technology, whereas Liqui-Pellet uses Liqui-Mass system and is part of the Liqui-Mass technology (1, 2, 5, 7).

The Liqui-Mass system’s essential difference from liquisolid system is that it is mostly a non-flowable wet mass admixture of active pharmaceutical ingredient (API) and excipients, whereas liquisolid system only includes dry looking nonadherent, free-flowing and readily compressible powder admixtures (8). This critical aspect of Liqui-Mass system leads to design of a system that is capable of generating versatile dosage form and a higher level of potential formulation modifications than liquisolid technology. 

It should be noted that although the Espindola *et al.* study (9) mentioned that they made a liquisolid pellet, it does not fall under the definition of liquisolid system, but rather resembles the Liqui-Mass system. Care should be taken when distinguishing liquisolid technology and Liqui-Mass technology as documented in a commentary article (5), where both technologies may at first glance appear the same (10), but in fact are not. 

The authors’ previous reported studies show that high liquid load factor does not pose a major flowability issue in the Liqui-Pellet. Therefore, it is possible to reduce the carrier and coating material required; thus, giving the potential for the addition of functional excipients without making the dosage form too heavy and bulky for swallowing (1–4). This, however, is not achievable in the liquisolid technology. Previous studies on Liqui-Pellet demonstrated that sodium bicarbonate (NaHCO_3_) of up to 42% w/w was added into a Liqui-Pellet formulation to enhance drug release rate via an effervescent system, which promotes disintegration and the disruption of the stagnant layer (11), whilst maintaining a good dosage form size for swallowing (3). 

Some additional key advantages of Liqui-Mass technology are simplistic manufacturing approach, the low cost of manufacturing, use of green technology, the potential for easy upscale of production and use of common and easily obtainable excipients (1–4). 

Technologies such as 3-D printing of drugs hold a greater risk to companies due to the introduction of new machinery and tailored regulation, which can disrupt the current working system and present new risks. The Liqui-Pellet technology uses machinery and excipients commonly found in a pharmaceutical companies’ manufacturing facility, which means risks and disruption to the current working system are kept to a minimum. In addition, flow property is crucial for smooth large-scale manufacturing, which Liqui-Pellet has no problem with. Poor flow property could lead to problem with drug uniformity due to non-uniform filling of capsule or die in tableting machines. 

Oral drug bioavailability is primarily affected by a drugs’ solubility profile, dissolution rate and permeability (12). In terms of the physicochemical aspect, drug dissolution rate and the solubility of water-insoluble drugs are the major reasons for the poor bioavailability of BCS Class II drugs (13). This is because the dissolution rate is often the rate-limiting step for the absorption of such drugs (13). The mechanism by which Liqui-Pellets achieve enhanced drug release without the addition of functional excipients is similar to that of liquisolid formulations. It is thought that an increase in the dissolution rate of drugs from liquisolid formulations is mainly attributed to the large surface area of the molecularly dispersed drug now available for dissolution, increase of drug’s solubility at the microenvironment and improved wetting properties due to water-miscible liquid vehicle acting as a wetting agent on liquisolid primary particle or on potentially precipitated crystal (14, 15).

Among the different pelletization technologies, extrusion-spheronization is favoured for making a Liqui-Pellet as it is the most popular method of making pharmaceutical pellets (16, 17). In brief, extrusion-spheronization involves four key steps: (i) dry mixing, then granulation to produce a wet mass (18,19); (ii) shaping of the wet mass into a cylindrical shape in a process called extrusion (18, 19); (iii) cutting and moulding the extrudate into spheroid via a spinning friction plate with characteristic grooved surfaces in a process called spheronization (18–22) (iv) drying of the pellet, which encompasses oven-drying (23), fluidized bed drier (24, 25), microwave-drying (23), freeze-drying (26) and desiccation with silica-gel (27, 28). It should be appreciated that each of these processes has its own parameters, which can affect the pellet’s physicochemical properties. 

In a study reported previously (3), the authors incorporated NaHCO_3_, which is an effervescent agent, into the Liqui-Pellet formulation to increase the drug release rate. The effervescent agent promoted the disintegration of the microcrystalline cellulose (MCC)-based Liqui-Pellet, which is interesting because MCC-based pellet is known to be virtually non-disintegrating due to strong bonding within its structure (29, 30). In the current study, an attempt was made to further improve the effervescent Liqui-Pellet drug release rate by considering various parameters including; polysorbate 80 concentration, water content and the presence or absence of neusilin US2 as part of the new binary carrier mixture approach. In general, increasing the non-volatile co-solvent content to a maximum limit should increase API in its solubilized state, and reducing the water content to a minimum limit should reduce pellet bonding force in order to promote pellet disintegration (3, 4). Neusilin US2 that is a material with a large specific surface area (SSA) is used in combination with MCC as a carrier material to investigate its effect on the Liqui-Pellet physicochemical properties.

## Materials and Methods

***Materials***

Naproxen was acquired from TCI Co (Japan). Carrier materials used included Avicel PH-101, (FMC corp., UK) and amorphous form of magnesium aluminometasilicate (Neusilin US2) (Fuji Chemicals, Japan). The coating material used was hydrophilic fumed silica (Aerosil 300) (Evonik Industries AG, Hanau, Germany). Disintegrant and pH-modulating agents used were primojel (DFE Pharma, Goch, Germany) and sodium bicarbonate, (Acros, New Jersey, USA), respectively. Non-volatile co-solvent used was polysorbate 80 (Acros, Netherlands), which is also known as Tween 80. All other reagents and solvent used in the current research were of analytical grades. 

***Production of rapid disintegrating naproxen Liqui-Pellet***

The production of Liqui-Pellets and physical mixture pellets were all prepared in a similar manner; however, there are several parameters such as carrier composition, water content, non-volatile co-solvent concentration and liquid load factor (Table 1). Initial mixing of API and non-volatile co-solvent was carried out using a mortar and pestle. This produces the liquid medication, which then was blended into a specified carrier material. Additional excipients such as 32% w/w NaHCO_3 _(effervescent agent) and a specified amount of sodium starch glycolate (primojel, a superdisintegrant) was added into the formulation (Table 1). Note that 32% w/w NaHCO_3 _was used as initial studies suggested this was the most suitable concentration when considering dosage form weight and drug-releasing performance (3). The admixture was mixed for 2 min using the mixer function in the Caleva Multitab machine at a constant rate of 125 rpm. The primojel was added intra-granularly as previous studies also suggested that this was more effective at promoting disintegration than extragranular incorporation (2). A required amount of deionized water was added gradually to achieve the suitable rheological property of wet mass, which is critical for successful extrusion (Caleva Multitab, UK). The admixture was further blended for 5 min. Silicon dioxide (Aerosil 300; a coating material) was then incorporated into the wet mass and further blended for 5 min before being extruded. Following the extrusion process, spheronization was employed at an almost constant rotation of 4000 rpm, which was decreased to 2000 rpm if formulation seemed likely to agglomerate. The wet pellets were dried overnight in an oven, which was set at a constant temperature of 40 ^°^C.

The physical mixture pellet was produced in a similar manner as for Liqui-Pellet, except non-volatile co-solvent was not added. All formulation’s carrier to coating material ratios were kept constant at 20:1, respectively.

***Evaluation of rapid disintegrating Liqui-Pellet and physical mixture formulation***

*Flowability test studies*

To measure the flowability of the Liqui-Pellet formulations, three parameters, mainly flow rate (g/s), angle of repose (°) and Carr’s index (%) were studied. The flow rate was measured using a Flowability tester by recording the weight (g) and time (sec) of pellets flowing through a 10 mm diameter orifice. The Angle of repose was measured by placing a few grams of the specified formulation in a funnel with a 10 mm orifice diameter. The formulation flowed onto a 100 mm diameter circular test platform. The height of the heap of the pellets on the platform was then measured and the angle of repose was calculated using equation 1 (Eq. 1). To calculate Carr’s index, the bulk (P_b_) and tapped (P_t_) densities (100 taps) were determined by equation 2 (Eq. 2). All measurements were conducted in triplicate. 

Angle of repose = tan^-1^ (height of heap of sample/radius of heap of sample) (1) 

 CI% = (P_t_ – P_b_)/P_t_ × 100 

(2)

*Particle size measurement using sieve method*

Particle size analysis was carried out on all formulations using the sieve method. Five grams of each formulation was placed in a nest of sieves (Test sieve, Retsch, Germany) with different aperture sizes of 2000, 1000, 850, 500 and 250 µm. The sieve was then placed on a mechanical shaker (AS 200, Retsch, Germany), which vibrated for 5 minutes. The pellet size distribution for the various formulations was determined based on the amount of solid mass left on each sieve and presented as percentages relative to the total mass.

*Robustness of formulated pellets via friability test*

Friability test was carried out for all formulations to find out whether they were robust enough for packaging and handling. Three grams of a specified formulation was placed in a friabiliator (D-63150, Erweka, Germany) along with 3 g of glass beads. The friabilator container was sealed in order to prevent pellets from leaving the rotating drum. The friabilator was set to 100 cycles at a speed of 25 rpm. The pellets were emptied from the container, dusted and their final weight was determined. The percentage weight loss was then calculated. 

*In vitro drug release test*

All Liqui-Pellet formulations were incorporated in a capsule size of 0, and their dissolution behaviour at either pH 1.2 (HCl buffer solution) or pH 7.4 (phosphate buffer solution) was investigated according to the USP method. Each capsule contained either physical mixture pellet or a specified Liqui-Pellet formulation equivalent to 25 mg of naproxen. The volume of the dissolution medium was 900 ml and the temperature was set up at 37.3±0.5 ^°^C. USP apparatus II (paddle method) was used at the agitation of 50 rpm. At different time intervals, samples from the dissolution vessels were automatically pumped into a UV spectrophotometer, and the absorbance of the samples was taken at 271 nm. 

To compare two dissolution profiles, two parameters namely the difference factor (f_1_) (Eq. 3) and similarity factor (f_2_) (Eq. 4) were employed (31). These two factors have been recommended by the US FDA (32).The FDA has placed more emphasis on the meaningful comparison of dissolution profile data. For example, the FDA scale-up and post-approval changes-modified release (SUPAC- MR. An in-depth explanation of these equations can be found in various literature (29, 33–36).The FDA has placed more emphasis on the meaningful comparison of dissolution profile data. For example, the FDA scale-up and post-approval changes-modified release (SUPAC- MR6). In general, the difference factor between 0-15 and the similarity factor between 50-100 indicate that two dissolution profiles are not different. In equations 3 and 4, the *n* represents the number of dissolution sample times and *R*_t _(reference) & *T*_t_ (test) represent the mean % of drug dissolved at each time point (*t*). 

f_1_= {[∑^n^_t=1_ R_t_ -T_t_ ]/[∑^n^_t=1_ R_t_]} •100 

(3)

f_2_ = 50•log {[1+ (1/n) ∑ ^n^_t=1 _(R_t_-T_t_)^2^]^-0.5^•100} 

(4)

***Stability studies***

Accelerated stability tests were conducted on formulations F-6 and F-7 (chosen as they had the fastest dissolution rate). The storage condition was set at 40 °C with a relative humidity of 75%, lasting for 3 months. Drug dissolution profiles were recorded each month of the 3 months.

***Tomographic studies***

In this study, only formulations F-6 and F-7 were analysed using X-ray microtomography (XμT), (Nikon XT H 225, Nikon Corp. Tokyo, Japan). Formulations F-6 and F-7 were used because they displayed the fastest dissolution rate. The instrument was set up using a tungsten target, with 90 kV accelerating voltage and an 80 μA gun current. Formulations F-6 or F-7 was mounted using a double-sided adhesive tape onto a sample stage. After this setup, a set of 1583 projections was collected from the instrumentation after which these images were reconstructed using CT-Pro, and then examined using a VG Studio 2.1 software (36, 37). 

## Results

***Micromeritics and pharmaceutical properties of novel effervescent Liqui-Pellets***

**Figure 1:**
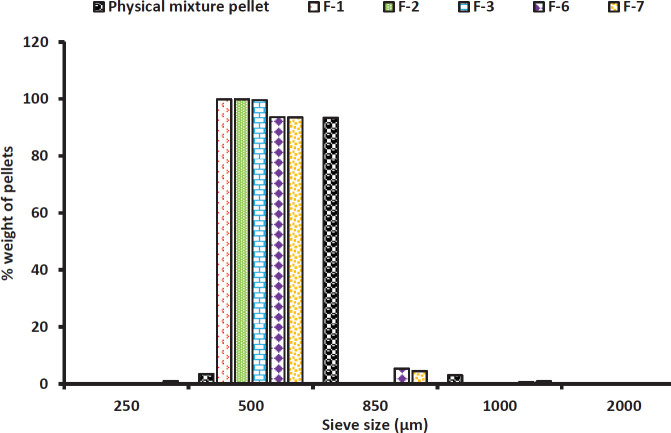
The distribution of particle size of all formulations

The micromeritics properties of the obtained Liqui-Pellet formulations are shown in Table 2 and Figure 1. According to flowability studies (Table 2), all formulations showed excellent, excellent to good or good flow properties. The particle size distribution of all the formulations shown in Figure 1 reveals that all the formulations show a narrow size distribution. Apart from the physical mixture pellet, all formulations mainly fall into the size of 500 µm.

Friability results showed that the percentage weight loss of all formulations after being treated in the friabilator were all below 1% (0.16 -0.98%). From the time this investigation was carried out, there is no standard friability test on pellets; therefore, USP standards for friability test on tablets was adapted, meaning weight loss of less than 1% being acceptable. 

**Figure 2:**
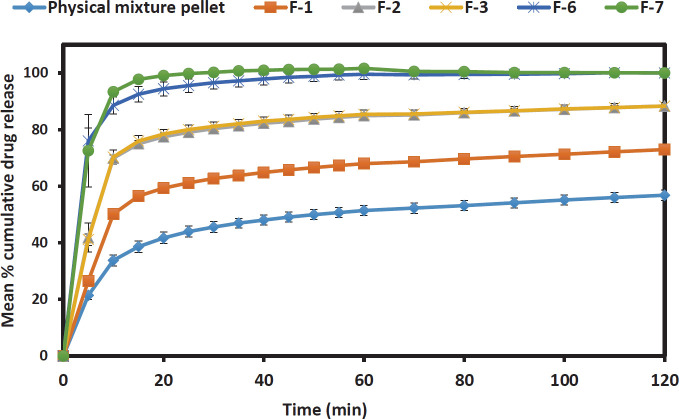
Dissolution profile of physical mixture pellet capsule and all successful Liqui-Pellet formulations capsule at pH 1.2

**Figure 3:**
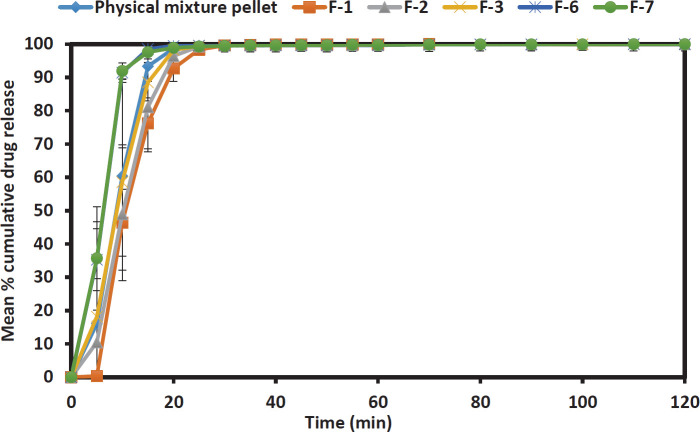
Dissolution profile of physical mixture pellet capsule and all successful Liqui-Pellet formulations capsule at pH 7.4

Dissolution profiles of various Liqui-Pellet formulations carried out at pH 1.2 and 7.4 are shown in Figure 2 and Figure 3, respectively. The optimized effervescent Liqui-Pellet formulations F-7 and F-6 showed a remarkably enhanced drug release profile at pH 1.2 (Figure 2), where naproxen is practically insoluble in such acidic pH (solubility of 27 mg/l (39)). In formulation F-7, the drug release was extremely fast and was nearing the 100% mark at 10 min (pH 1.2), which is similar to its drug dissolution profile at pH 7.4 as shown in Figure 3, where naproxen is freely soluble at this pH (solubility of 3347 mg/l) (38). 

The drug dissolution profile of formulations F-6 and F-7 (Figure 5 and Figure 6 respectively) were further investigated at pH 1.2 to observe if the stressed condition of the accelerated stability test affects the drug release rate. The results showed that the effect of storage time on the drug release from aged Liqui-Pellet is not hugely different from the freshly made Liqui-Pellets, which is discussed in the discussion section below.

## Discussion

***Production of rapid disintegrating naproxen Liqui-Pellet***

Liqui-Pellet formulations were all successfully produced except for formulations F-4 and F-5. Formulation F-4 had the highest amount of polysorbate 80 (27% w/w) and F-5 had the highest amount of water (6.4 ml per 20 g Liqui-Mass composition) in the Liqui-Pellet dosage form. Polysorbate 80 and water content increases the extrudate’s plastic property (3). Formulations F-4 and F-5 extrudate’s plastic property increased beyond the acceptable limit, causing agglomeration during the spheronization process, hence, leading to formulation failure. The acceptable limit is the range that would not cause agglomeration and will produce pellets. This reflects the importance of understanding the ideal extrudate’s plastic property and the parameters that affect it, as water and non-volatile co-solvent content have a major influence in an extrudate’s plastic property, which in turn can determine the success of Liqui-Pellet production (3). 

***Micromertics property of effervescent Liqui-Pellets***

The smooth flow of Liqui-Pellet is one of the key features that makes it a suitable commercial product. Liqui-Pellets can achieve high liquid load factors and yet have excellent flow properties without requiring a considerable addition of carriers and coating materials (Table 2). Such features allow the Liqui-Pellet to outperform liquisolid formulation in terms of enhanced dissolution rate, flow properties and versatility in formulation modification such as the addition of functional excipients (4). 

The current study showed that Liqui-Pellet technology is capable of producing narrow size distribution (Figure 1). This narrow size distribution is also observed rather consistently by various studies on Liqui-Pellet (1, 7), indicating that the use of extrusion-spheronization technology for Liqui-Pellet production is commercially practical. The narrow size distribution makes the handling of these Liqui-Pellets more ideal, for example, capsule filling and the reduced risk of failing dosage form uniformity of content quality control test. 

***Pharmaceutical properties of effervescent Liqui-Pellets***

Although F-3 and F-7 passed the friability test, it can be observed that they have the highest amount of weight loss (0.94% and 0.98%, respectively), which suggests that they are less robust than the other formulations. This is most likely due to F-3 and F-7 having the highest amount of polysorbate 80 possible for successful production of this particular Liqui-Pellet (23% w/w). Note that F-4 (27% w/w polysorbate 80) does not count as this formulation failed to produce pellets. With a high amount of polysorbate 80, less water is required in the formulation for successful extrusion and spheronization. The reduction in water leads to a decrease in the cohesive force within the F-3 and F-7 formulations; thus, they are less robust. Water content seems to be an important factor affecting MCC-based pellet as stated in various literature (39–41). Nonetheless, all formulations passed the friability test; and as such, there is more room for formulation optimization for improvement of effervescent Liqui-Pellet robustness. 

Formulation F-7 has been optimized by using a high amount of polysorbate 80 (23% w/w) and a lower amount of water content (3.21 ml per 20 g of Liqui-Mass admixture) that was possible to produce Liqui-Pellet and not resulted to agglomeration. These two parameters have been reported to affect the Liqui-Pellet drug release profile substantially in a previous study (4). Although polysorbate 80 and the water content are optimized, the single most significant factor resulting in F-7 remarkable rapid and explosive drug release other than NaHCO_3_ is the use of neusilin US2 in the formulation. Neusilin is considered as a multifunctional excipient and is known as an excellent absorbent material (43) with disintegrant and suspending properties (11). Perhaps such properties promote fast disintegration in F-6 and F-7, leading to a rapid drug release rate.

Despite F-7 having a lower concentration of NaHCO_3_ (32% w/w) than a naproxen effervescent formulation from a previous study, where NaHCO_3_ concentration of 42% w/w was used (4), F-7 interestingly has a significantly faster drug release rate under acidic condition of pH 1.2 (f_1_= 79.26 and f_2_= 26.16). This indicates that although NaHCO_3_ concentration is 10% w/w lower in F-7, the presence of neusilin US2 resulted in a remarkable enhancement of drug release that even surpasses a similar formulation with 10% more NaHCO_3_ but without neusilin US2.

To further appreciate such improvement in the enhanced drug release of F-7, it is noteworthy to compare this naproxen effervescent Liqui-Pellet with a current naproxen liquisolid formulation. In Tiong and Elkordy’s study, the best naproxen liquisolid tablet formulation obtained ~60% drug release in 1 hr (44), whereas F-7 neared 100% in 10 min. Such drug dissolution profile along with excellent flowability (Table 2), narrow size distribution (Figure1) and acceptable size and weight for swallowing, indicates Liqui-Pellet potential as a promising next-generation oral dosage form with capability for rapid drug release. Even when comparing F-7 Liqui-Pellets performance with other promising technology such as solid dispersions, the Liqui-Pellet displays a superior enhanced drug release. Naproxen (20 mg) solid dispersion formulation in Adibkia, Barzegar-Jalali, *et al.* study (45), reached 100% drug release rate at about 2 hr even though the dissolution tests were at pH 3 where naproxen is more soluble than in pH 1.2, which was used for Liqui-Pellet. 

Data from Figure 2 shows that F-6 has a rapid drug dissolution profile similar to F-7 (f_1_= 5.05 and f_2_= 72.3). Despite F-6 having a lower amount of polysorbate 80 (19% w/w) than F-7 (23% w/w), F-6 achieved fast dissolution rate similar to F-7 due to neusilin US2 in the formulation. Neusilin US2 appears to have a major influence on the drug release. It is not clear why this is the case but it could be due to neusilin US2 unique property of high specific surface area influencing the overall physical property of the formulation.

It is observed that an increase in polysorbate 80 and a reduction of water content increases the drug release rate. This is shown in Figure 2 where F-2 (containing 21% w/w polysorbate 80 and 3.2 ml of water per 20 g of Liqui-Mass admixture) shows a faster dissolution rate than F-1 (containing 19% w/w polysorbate 80 and 5.6 ml of water per 20 g of Liqui-Mass admixture) by ~15 % after 2 hr (f_1_= 21.22 and f_2_=38.95). However, as polysorbate 80 concentration further increases and water content further decreases, their influence on the drug dissolution rate diminishes. This can be observed in F-3 (containing 23% w/w polysorbate 80 and 3.12 ml of water per 20 g of Liqui-Mass admixture) and F-2 (containing 21% w/w polysorbate 80 and 3.2 ml of water per 20 g of Liqui-Mass admixture), where their dissolution profile is very similar (f_1_= 0.64 and f_2_= 96.78), despite the difference in polysorbate 80 concentration and water content.

**Figure 4:**
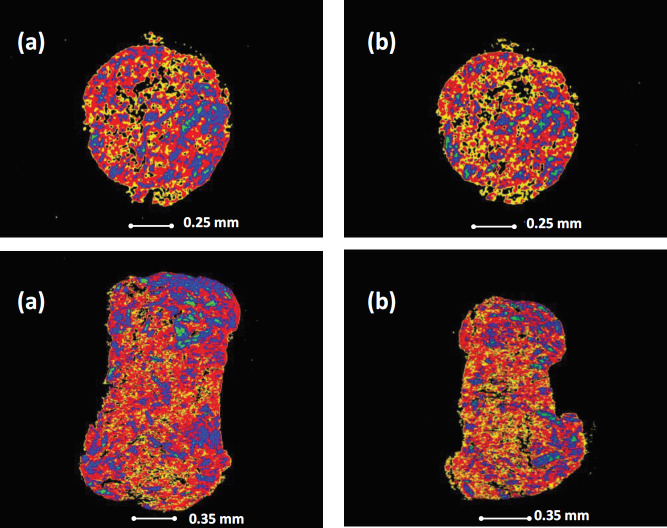
X-ray microtomographic sagittal and diametric images of Liqui-Pellet formulations of (a) F6 (b), and F7 detailing the homogeneity of the formulation mix of the Liqui-Pellet technology. Please see Table 1 for the composition of F6 and F7

Dissolution test results under pH 7.4 (Figure 3) show that F-6 and F-7 have the fastest drug release rate. Using XμT to further understand F-6 and F-7 show that they are very similar (based on the constituents of the formulations) (Figure 4). The XμT technique that is based on the differential absorbance of X-rays between materials of differing electron density (45, 46) allows the differentiation of the different materials used. The reconstructed X-ray microtomographic images using the CT-Pro and VG Studio 2.1 software showed the Liqui-Pellet to be very uniform. This was very evident in the sagittal and diametric cross-sectional images from the XμT (Figure 4). The similarity in the images despite their difference in the polysorbate content can also account for their similar drug release patterns observed in pH 1.2 and 7.4. It was also observed that the drug release rate improves with increasing polysorbate 80, for example, F-3 (23% w/w polysorbate 80) is better than F-2 (21% w/w polysorbate 80), and F-2 is better than F-1 (19% w/w polysorbate 80). It was interesting to observe the physical mixture pellet having a slightly better-enhanced dissolution profile than F-1, F-2 and F-3 in Figure 3. This is due to naproxen being freely soluble in pH 7.4 and also the use of the NaHCO_3_ may further enhance the alkaline pH; thus, the liquid vehicle plays a less vital role in drug dissolution as naproxen is already freely soluble in this environment. Overall, the optimized naproxen effervescent Liqui-Pellet is capable of remarkable drug release enhancement, with NaHCO_3_ and neusilin US2 being a major contributor to this observation. 

***Accelerated stability studies***

Comparing the dissolution profile of fresh formulation F-6 (month 0) and aged F-6 (month 1) shows a difference in the dissolution profile (f_1_ = 27.93 and f_2_ = 34.66). This difference in dissolution profile may be due to changes in the formulation over the storage time such as the pellet becoming harder over time due to further water loss via evaporation. Also, there may have been potentially a small degree of recrystallization of naproxen within the first month. It should be noted that sodium bicarbonate can intensify the darkening of salicylate and temperature and humidity can affect its stability (11).

The reduction in drug release becomes less apparent after this first month where F-6 dissolution profile in 1^st^ and 2^nd^ months gives f_1_ = 2.44 and f_2_ = 83.66 respectively. This indicates no significant difference in the dissolution profile. A similar observation was made between month 2 and month 3 where f_1_ and f_2_ are 3.23 and 73.60, respectively.

The stability test for formulation F-7, as shown in Figure 6, shows no significant differences in the dissolution profile between month 0 to month 1 (f_1_ = 6.94 and f_2_ = 60.96), month 1 to month 2 (f_1_ = 2.44 and f_2_ = 83.66) and month 2 to month 3 (f_1_ = 3.23 and f_2_ = 73.60). 

**Table 1:** Composition, parameters and production outcome of all formulations

**Formulation**	**Water content during extrusion-spheronization (ml) per 20g of admixture of API and excipient**	**Polysorbate 80 concentration (% w/w)**	**Liquid load factor**			**Primojel (mg)**	**Carrier composition**	**Mass of carrier material (Avicel-PH101) (mg)**	**Mass of coating material (Aerosil 300) (mg)**	**Successfully spheronized into pellet? (Yes/ No)**	**Total weight of pellets equivalent to 25mg naproxen (mg) **
Physical mixture pellet	7.00				5.91		Type 1^a^	58.06	2.90	Yes	135.25
F-1	5.60	19		1	5.92		Type 1	62.54	3.15	Yes	197.20
F-2	3.21	21		1.14	5.92		Type 1	58.06	2.90	Yes	197.20
F-3	3.12	23		1.23	5.92		Type 1	55.06	2.75	Yes	197.20
F-4	1.60	27		1.65	5.92		Type 1	47.55	2.37	No	197.20
F-5	6.40	19		1	5.92		Type 2^b^	62.54	3.15	No	197.20
F-6	3.20	19		1	5.92		Type 2	62.54	3.15	Yes	197.20
F-7	3.20	23		1.23	5.92		Type 2	55.06	2.75	Yes	197.20

Note that all formulations contain 25 mg of naproxen, and 32% w/w NaHCO3, and the carrier to coating material is at a ratio of 20:1

Type 1- 100% avicel PH101

Type 2- 50% avicel PH101 & 50% neusilin US2

**Table 2:** Flow rate (g/sec), Angle of repose and Carr’s compressible index (CI%) of all formulations (n=3)

**Formulation** ^a^	**Flow Rate (g/sec) ± SD** ^b^	**Angle of repose ± SD** ^b^	**CI% ± SD** ^b^	**Inference according to Angle of repose**	**Inference according to CI%**
**Physical mixture pellet **	8.75 ± 0.19	24.39 ± 0.56	13.32 ± 0.00	Excellent flowability	Good flowability
**F-1**	8.10 ± 0.17	26.71 ± 0.20	10.23 ± 0.00	Excellent flowability	Excellent to good flowability
**F-2**	8.12 ± 0.27	27.32 ± 0.44	10.33 ± 0.57	Excellent flowability	Excellent to good flowability
**F-3**	7.81 ± 0.28	28.92 ± 0.49	10.33 ± 1.14	Excellent flowability	Excellent to good flowability
**F-6**	7.86 ± 0.19	28.58 ± 1.00	11.17 ± 0.00	Excellent flowability	Good flowability
**F-7**	8.37 ± 0.11	26.83 ± 0.79	10.23 ± 0.00	Excellent flowability	Excellent to good flowability

^a^ For the composition of each formulation refer to Table 1

^b^ SD, standard deviation from the mean

**Figure 5:**
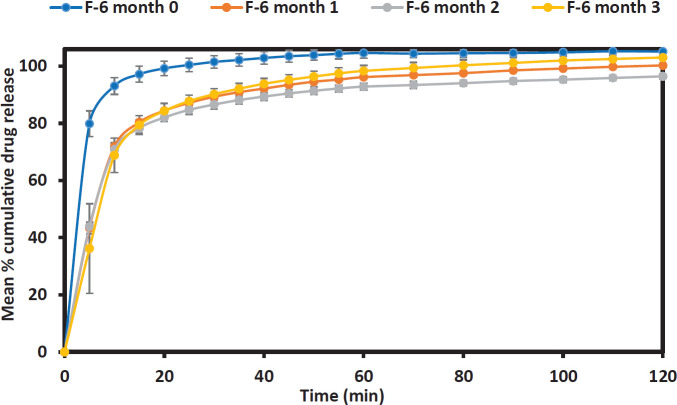
Stability test of formulation F-6 represented through dissolution profile taken each month over the period of 3 months under pH 1.2

**Figure 6:**
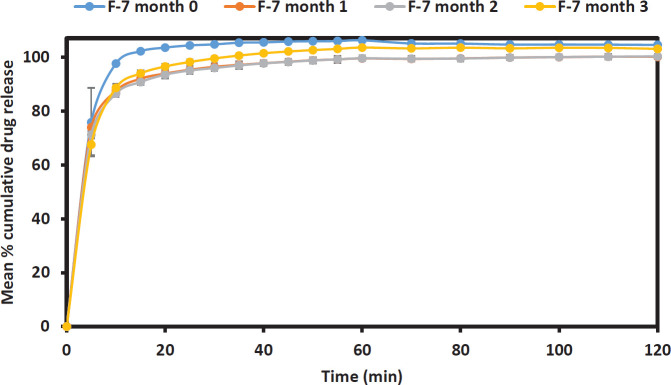
Stability test of formulation F-7 represented through dissolution profile taken each month over the period of 3 months under pH 1.2

## Conclusion

This study has proven that Liqui-Mass technology is capable of producing explosive and rapid drug release. The data from the dissolution test shows that naproxen Liqui-Pellets can achieve 100% drug release within 20 min at an acidic pH of 1.2, which naproxen is known to be practically insoluble at. The results suggest that the key factor contributing to this remarkable drug release profile is the ability for the Liqui-Pellet to support sufficient effervescent agent (NaHCO_3_) and neusilin US2 in the formulation. Furthermore, the accelerated stability test shows that some of the formulations maintained their original dissolution behaviour over the 3 months. These results, therefore, seem to be more superior than naproxen in liquisolid formulation or even other promising and competitive technology such as naproxen in solid dispersion systems. This technology thus displays the exciting potential of the Liqui-Pellet being a valuable next-generation dosage form for the future. 

## Funding Source

This research did not receive any specific grant from funding agencies in the public, commercial, or not for profit sectors.
